# Clinical Symptom Burden and Its Association With Echocardiographic Parameters and Comorbidities in Heart Failure With Preserved Ejection Fraction: A Retrospective Study

**DOI:** 10.7759/cureus.118136

**Published:** 2026-10-18

**Authors:** Reena A Athisivam, Venkkatesh Nagajothi Shanmugasundaram, Syed Abrar Hasan Syed Javid Hasan, Mohit Kumar Ramkumar, Neha V Damodar, Neeharikka Gopalakrishnan, Deviabhirami N S, Horavaasan H, Hari Vishak Pandian Jeyamurugan, Hariharan Srinivasan

**Affiliations:** 1 Internal Medicine, Peripheral Government Hospital, Periyar Nagar, Chennai, IND; 2 Psychiatry, Hospital for Mental Health, Vadodara, Vadodara, IND; 3 Internal Medicine, Raigmore Hospital, Inverness, GBR; 4 General Surgery, Government Villupuram Medical College and Hospital, Mundiyampakkam, IND; 5 Surgery, King Edward Memorial Hospital and Seth Gordhandas Sunderdas Medical College, Mumbai, IND; 6 Internal Medicine, Fatima University Medical Center, Valenzuela, PHL; 7 Anesthesiology, Siddhartha Medical College, Vijayawada, IND; 8 Anatomy, Siddhartha Medical College, Vijayawada, IND; 9 General Practice, Aster Whitefield Hospital, Bangalore, IND; 10 General Practice, Zahran Operations &amp; Maintenance, Jeddah, SAU

**Keywords:** echo cardiogram, heart failure, heart failure with preserved ejection fraction (hfpef), lv ejection fraction (lvef), sleep dyspnea

## Abstract

Introduction: Heart failure with preserved ejection fraction (HFpEF) is a heterogeneous clinical syndrome in which substantial symptoms may occur despite preserved left ventricular ejection fraction (LVEF). Echocardiographic abnormalities involving ventricular filling, left atrial remodeling, diastolic function, and pulmonary pressures may contribute to symptom burden. This study evaluated the association between clinical symptom severity, echocardiographic parameters, and comorbidities in patients with HFpEF.

Study: This retrospective observational cross-sectional study was conducted at the Government Peripheral Hospital, Periyar Nagar, Chennai, Tamil Nadu, from March 2025 to March 2026. A total of 216 patients aged ≥18 years with HFpEF and LVEF ≥50% were included. Clinical symptom burden was assessed using New York Heart Association (NYHA) functional class and categorized as I-II or III-IV. Demographic characteristics, comorbidities, and echocardiographic parameters, including LVEF, left ventricular mass, left atrial volume index (LAVI), E/e′ ratio, diastolic dysfunction grade, and estimated pulmonary artery systolic pressure (PASP), were evaluated. Multivariable logistic regression identified factors independently associated with NYHA III-IV symptoms.

Results: The mean age was 67.8 ± 10.4 years, and 53.7% were female. Hypertension was the most prevalent comorbidity (80.6%), while 76.9% had ≥2 major comorbidities. Fifty-five patients (25.5%) had NYHA III-IV symptoms. Compared with NYHA I-II, the severe symptom group had higher E/e′ (17.4 ± 5.0 vs. 12.6 ± 4.0), LAVI (44.5 ± 12.1 vs. 34.2 ± 9.1 mL/m²), LV mass, and PASP (43.9 ± 12.4 vs. 36.8 ± 8.7 mmHg), with significantly more advanced diastolic dysfunction. On multivariable analysis, E/e′ (adjusted OR 1.18), LAVI (adjusted OR 1.05), and PASP (adjusted OR 1.04) were independently associated with severe symptoms.

Conclusion: Symptom severity in HFpEF was associated more strongly with abnormal filling pressures, left atrial remodeling, and pulmonary hemodynamics than with preserved systolic function alone. E/e′, LAVI, and PASP may provide useful complementary markers of symptomatic burden and support a multidimensional echocardiographic assessment of HFpEF.

## Introduction

Heart failure with preserved ejection fraction (HFpEF) is a common and increasingly recognized form of heart failure, characterized by clinical symptoms and signs despite a preserved left ventricular ejection fraction (LVEF), generally defined as ≥50% [1]. HFpEF represents a heterogeneous clinical syndrome in which cardiovascular and metabolic comorbidities, including hypertension, obesity, diabetes mellitus, atrial fibrillation, and chronic kidney disease, contribute substantially to disease development, clinical presentation, and functional limitation [2]. Unlike heart failure with reduced ejection fraction, the relationship between left ventricular systolic function and symptom severity in HFpEF is less straightforward, making clinical assessment and phenotyping particularly challenging. 

Echocardiography plays a central role in the evaluation of HFpEF by providing information regarding cardiac structure and diastolic function. Parameters such as left atrial volume, left ventricular mass, mitral inflow and annular velocities, E/e′ ratio, tricuspid regurgitation velocity, and estimated pulmonary artery pressure provide indirect evidence of abnormal filling pressures and cardiac remodeling [2]. However, individual echocardiographic parameters may not consistently correlate with the severity of symptoms, particularly because HFpEF is influenced by multiple extracardiac and cardiovascular comorbidities [3].

Understanding whether greater clinical symptom burden is associated with specific echocardiographic abnormalities or with the accumulation of comorbid conditions may improve clinical phenotyping and risk assessment. Therefore, the present study aims to evaluate the association between clinical symptom burden, echocardiographic characteristics, and common cardiovascular and metabolic comorbidities among patients with HFpEF.

## Materials and methods

This retrospective observational cross-sectional study was conducted at the Government Peripheral Hospital (affiliated to Madras Medical College), Periyar Nagar, Chennai, Tamil Nadu, over a one-year period from March 2025 to March 2026. The study aimed to evaluate the association between clinical symptom burden and echocardiographic parameters among patients with HFpEF and to determine whether common cardiovascular and metabolic comorbidities were associated with greater symptom severity. The primary objective was to assess the relationship between clinical symptom burden, particularly the New York Heart Association (NYHA) functional class, and echocardiographic parameters of cardiac structure and diastolic function. Secondary objectives were to describe the demographic and clinical characteristics of patients with HFpEF; determine the prevalence of major comorbidities including hypertension, diabetes mellitus, coronary artery disease, atrial fibrillation, chronic kidney disease, and obesity; assess the association of symptom burden with left atrial size or volume, left ventricular mass or hypertrophy, E/e′ ratio, grade of diastolic dysfunction, LVEF, and estimated pulmonary artery systolic pressure (PASP); and identify clinical and echocardiographic factors independently associated with greater symptomatic burden.

Patients aged ≥18 years with a documented clinical diagnosis of HFpEF, LVEF ≥50%, clinical symptoms and/or signs compatible with heart failure, and an available echocardiographic assessment during the study period were eligible for inclusion. HFpEF was defined for this retrospective study as a clinical syndrome of heart failure with an LVEF of ≥50%, supported by the review of available clinical records and echocardiographic reports for findings consistent with cardiac structural or functional abnormalities. The assessment considered documented symptoms and/or signs of heart failure, left ventricular systolic function, and available echocardiographic evidence of structural abnormalities, diastolic dysfunction, or elevated filling pressures. LVEF ≥50% was not considered sufficient in isolation to establish the diagnosis. Patient eligibility was determined from the clinical and echocardiographic information available in the medical records. The HFA-PEFF diagnostic algorithm was not formally calculated for individual patients as part of this retrospective study. Diagnostic classification was based on the documented clinical diagnosis and review of the available clinical and echocardiographic records. Accordingly, the study did not undertake a systematic, score-based diagnostic adjudication of HFpEF, and the findings should be interpreted in the context of the diagnostic information available in routine clinical practice. Patients with LVEF <50%, significant primary valvular heart disease capable of independently explaining the symptoms, congenital heart disease, acute myocardial infarction at the index assessment, primary pulmonary hypertension as the principal cause of symptoms, constrictive pericarditis, infiltrative cardiomyopathy as the primary diagnosis, or inadequate medical records preventing assessment of the principal study variables were excluded. Eligible patients were identified from hospital medical records and echocardiography records, and all eligible records available during the predefined study period were considered for inclusion. Exclusion was based on the predefined eligibility criteria and documented evidence; additional investigations were not performed solely for the purposes of this retrospective study. Records that failed to meet the eligibility criteria were excluded before analysis. Records with incomplete information were excluded only when the missing information prevented confirmation of eligibility or assessment of the primary outcome.

Clinical, demographic, comorbidity, laboratory, and echocardiographic data were extracted retrospectively using a structured data-collection proforma. Demographic variables included age, sex, body mass index (BMI) and smoking status. Clinical variables included dyspnea, orthopnea, paroxysmal nocturnal dyspnea, pedal edema, fatigue, exercise intolerance, NYHA functional class, heart rate, blood pressure and oxygen saturation. NYHA functional class was used as the principal measure of symptom burden, and for the primary analysis, patients were categorized into NYHA class I-II and NYHA class III-IV. Major comorbidities recorded were hypertension, diabetes mellitus, coronary artery disease, atrial fibrillation, chronic kidney disease, obesity, dyslipidemia, chronic obstructive pulmonary disease, and anemia. Laboratory variables included hemoglobin, serum creatinine/eGFR, HbA1c or fasting glucose, and BNP or NT-proBNP when available. Echocardiographic variables included LVEF, left ventricular end-diastolic and end-systolic dimensions, LV mass or presence of LV hypertrophy, left atrial diameter or volume index, E/A ratio, e′ velocity, E/e′ ratio, grade of diastolic dysfunction, tricuspid regurgitation velocity, estimated PASP, and inferior vena cava diameter and respiratory variation where available. Echocardiographic measurements were obtained from the available clinical reports and were not independently reassessed by a second cardiologist as part of this study. The primary outcome was the association between NYHA functional class and echocardiographic markers of cardiac structure and diastolic function, particularly E/e′ ratio, left atrial size or volume, LV hypertrophy or mass, diastolic dysfunction, and estimated PASP. 

The sample size was calculated based on the anticipated correlation between clinical symptom severity and a principal echocardiographic parameter. Assuming an anticipated correlation coefficient of r = 0.20, a two-sided α of 0.05, and 80% power, the minimum required sample size was 194 participants using the Fisher z-transformation method [4]. To account for approximately 10% of records that could be incomplete or unsuitable for final analysis in a retrospective study, the planned minimum sample size was increased to 216 participants. A total of approximately 250 medical records were screened against the predefined eligibility criteria. Patients were excluded when the available clinical information suggested a plausible alternative explanation for breathlessness or when missing information prevented adequate assessment of eligibility or the primary study outcome. A total of 216 eligible patients were included in the final analysis, and recruitment ceased once the target sample size was reached.

Continuous variables were assessed for distribution and expressed as mean ± standard deviation or median with interquartile range, as appropriate. Categorical variables were presented as frequencies and percentages. Patients with NYHA class I-II and NYHA class III-IV were compared using the independent-samples t-test or Mann-Whitney U test for continuous variables and the chi-square test or Fisher's exact test for categorical variables, as appropriate. Multivariable binary logistic regression was performed to identify factors independently associated with severe symptom burden, defined as NYHA class III-IV. Echocardiographic parameters demonstrating statistically significant associations with symptom severity in the univariable comparisons, together with parameters considered clinically relevant, were considered for inclusion in the multivariable logistic regression model. The final model included selected echocardiographic predictors, with the aim of identifying parameters associated with NYHA class III-IV symptoms after adjustment for the other echocardiographic predictors included in the model. Demographic characteristics and clinical comorbidities were not included as adjustment variables. The selected echocardiographic predictors were considered together in the multivariable model. A formal collinearity diagnostic was not documented; therefore, potential collinearity between echocardiographic predictors should be considered when interpreting the model estimates. Results of logistic regression were expressed as odds ratios (ORs) with 95% confidence intervals (CIs). Missing data were not imputed; analyses were performed using available observations, and the number of participants contributing to individual analyses was reported where applicable. A two-sided p-value < 0.05 was considered statistically significant. The study was conducted in accordance with institutional ethical requirements and the principles of the Declaration of Helsinki. Reporting of the observational study was guided by the Strengthening the Reporting of Observational Studies in Epidemiology (STROBE) recommendations [4].

## Results

In the cohort of 216 patients with HFpEF, the mean age was 67.8 ± 10.4 years, with 139 (64.4%) patients aged ≥65 years. There were 100 (46.3%) males and 116 (53.7%) females. The mean BMI was 27.4 ± 4.6 kg/m², with obesity present in 78 (36.1%) patients. A history of smoking was present in 63 (29.2%) patients (Table 1).

**Table 1:** Demographic characteristics of the study population Data are presented as mean ± SD or n (%), as appropriate. BMI: body mass index; kg/m^2^: kilograms per square meter; SD: standard deviation; n (%): number of patients (percentage)

Demographic characteristic	Value (mean ± SD)/ n (%)
Age, years	67.8 ± 10.4
Age ≥65 years	139 (64.4%)
Male	100 (46.3%)
Female	116 (53.7%)
BMI, kg/m²	27.4 ± 4.6
Obesity	78 (36.1%)
Smoking history	63 (29.2%)

In the cohort, dyspnea was the predominant clinical manifestation, reported in 186 (86.1%) patients, followed by exercise intolerance in 151 (69.9%) and pedal edema in 112 (51.9%). Orthopnea and paroxysmal nocturnal dyspnea were present in 96 (44.4%) and 71 (32.9%) patients, respectively. NYHA functional class II was the most frequently observed category, while 55 (25.5%) patients had NYHA class III-IV symptoms, representing the severe symptom-burden group for subsequent analyses. The mean resting heart rate was 78.6 ± 12.1 beats/min, while the mean systolic and diastolic blood pressures were 137.4 ± 18.6 mmHg and 78.2 ± 10.4 mmHg, respectively (Table 2).

**Table 2:** Clinical characteristics of the study population Data are presented as mean ± SD or n (%), as appropriate. NYHA: New York Heart Association; SD: standard deviation; %: percentage; n: number of patients; mmHg: millimetres of mercury; beats/min: beats per minute

Clinical characteristic	Value
Dyspnea	186 (86.1%)
Orthopnea	96 (44.4%)
Paroxysmal nocturnal dyspnea	71 (32.9%)
Pedal edema	112 (51.9%)
Fatigue	127 (58.8%)
Exercise intolerance	151 (69.9%)
NYHA functional class	
NYHA I	24 (11.1%)
NYHA II	137 (63.4%)
NYHA III	44 (20.4%)
NYHA IV	11 (5.1%)
NYHA III–IV	55 (25.5%)
Heart rate, beats/min	78.6 ± 12.1
Systolic blood pressure, mmHg	137.4 ± 18.6
Diastolic blood pressure, mmHg	78.2 ± 10.4
Oxygen saturation, %	96.2 ± 2.1

Hypertension was the most prevalent comorbidity in the cohort, present in 174 (80.6%) patients, followed by diabetes mellitus in 101 (46.8%) and chronic kidney disease in 67 (31.0%). Coronary artery disease and atrial fibrillation were present in 58 (26.9%) and 47 (21.8%) patients, respectively. Obesity was documented in 78 (36.1%) patients. Overall, 166 (76.9%) patients had two or more major comorbidities, demonstrating a substantial multimorbidity burden within the simulated HFpEF population (Table 3).

**Table 3:** Cardiovascular and metabolic comorbidities in the study population n: number of patients; %: percentage

Comorbidity	n (%)
Hypertension	174 (80.6%)
Diabetes mellitus	101 (46.8%)
Obesity	78 (36.1%)
Chronic kidney disease	67 (31.0%)
Coronary artery disease	58 (26.9%)
Dyslipidemia	91 (42.1%)
Atrial fibrillation	47 (21.8%)
Chronic obstructive pulmonary disease	29 (13.4%)
Anemia	52 (24.1%)
Number of major comorbidities	
0–1 comorbidity	50 (23.1%)
≥2 comorbidities	166 (76.9%)

The echocardiographic profile demonstrated preserved left ventricular systolic function, with a mean LVEF of 61.4 ± 5.8%. Left ventricular hypertrophy was present in 118 (54.6%) patients, while left atrial enlargement was observed in 101 (46.8%). The mean E/e′ ratio was 13.8 ± 4.7, and grade II diastolic dysfunction was the most frequent grade. Elevated estimated PASP was present in 58 (26.9%) patients (Table 4). 

**Table 4:** Echocardiographic characteristics of the study population LVEF: left ventricular ejection fraction; LV: left ventricle; E/A: ratio of early to late ventricular filling velocities; E/e′: ratio of early transmitral flow velocity to early diastolic mitral annular velocity; e′: early diastolic mitral annular velocity; TR: tricuspid regurgitation; PASP: pulmonary artery systolic pressure; IVC: inferior vena cava; mm: millimetres; cm/s: centimetres per second; mL/m²: millilitres per square metre; g: grams; mmHg: millimetres of mercury; %: percentage; n: number of patients

Echocardiographic parameter	Value
LVEF, %	61.4 ± 5.8
LV end-diastolic diameter, mm	49.8 ± 5.4
LV end-systolic diameter, mm	32.4 ± 4.8
LV mass, g	187.6 ± 48.3
Left ventricular hypertrophy	118 (54.6%)
Left atrial diameter, mm	41.2 ± 5.6
Left atrial volume index, mL/m²	36.8 ± 10.7
E/A ratio	0.82 ± 0.31
E/e′ ratio	13.8 ± 4.7
Septal e′ velocity, cm/s	5.8 ± 1.6
Diastolic dysfunction grade	
Grade I	73 (33.8%)
Grade II	112 (51.9%)
Grade III	31 (14.4%)
TR velocity, m/s	2.72 ± 0.42
Estimated PASP, mmHg	38.6 ± 10.2
Elevated PASP	58 (26.9%)
IVC diameter, mm	17.2 ± 4.1

Patients with NYHA class III-IV symptoms demonstrated greater structural and diastolic abnormalities than those with NYHA class I-II symptoms. The mean E/e′ ratio was 17.4 ± 5.0 in the NYHA III-IV group compared with 12.6 ± 4.0 in the NYHA I-II group (p < 0.001), while the mean LAVI was 44.5 ± 12.1 mL/m² versus 34.2 ± 9.1 mL/m², respectively (p < 0.001). LV mass was also higher in patients with greater symptom burden (214.5 ± 55.7 g vs. 178.4 ± 42.6 g; p < 0.001), and LV hypertrophy was more frequent (69.1% vs. 49.7%; p = 0.015). The distribution of diastolic dysfunction grades differed significantly between the two NYHA groups (Pearson χ² = 22.289, df = 2, p < 0.001). Grade III diastolic dysfunction was present in 17 (30.9%) patients with NYHA III-IV symptoms compared with 14 (8.7%) with NYHA I-II symptoms, whereas grade I dysfunction was less frequent in the more symptomatic group (8 (14.5%) vs. 65 (40.4%)). The mean estimated PASP was also higher in the NYHA III-IV group (43.9 ± 12.4 mmHg vs. 36.8 ± 8.7 mmHg; p < 0.001). LVEF remained preserved in both groups, although it was modestly lower among patients with NYHA III-IV symptoms (59.7 ± 6.1% vs. 62.0 ± 5.5%; p = 0.009) (Table 5).

**Table 5:** Association of echocardiographic parameters with symptom severity ᵃIndependent-samples t test, ᵇChi-square test, ᶜPearson chi-square test NYHA: New York Heart Association; LVEF: left ventricular ejection fraction; LV: left ventricle; LAVI: left atrial volume index; E/A: ratio of early to late ventricular filling velocities; E/e′: ratio of early transmitral flow velocity to early diastolic mitral annular velocity; e′, early diastolic mitral annular velocity; TR: tricuspid regurgitation; PASP: pulmonary artery systolic pressure; mL/m²: millilitres per square metre; cm/s: centimetres per second; m/s: metres per second; mmHg: millimetres of mercury; g: grams; SD: standard deviation; n: number of patients; %: percentage; df: degrees of freedom

Echocardiographic parameter	NYHA I-II (n = 161)	NYHA III–IV (n = 55)	p value	Test statistic
LVEF, %	62.0 ± 5.5	59.7 ± 6.1	0.009ᵃ	t = 2.603
LV mass, g	178.4 ± 42.6	214.5 ± 55.7	<0.001ᵃ	t = −4.997
LV hypertrophy, n (%)	80 (49.7%)	38 (69.1%)	0.013ᵇ	χ² = 6.226
LAVI, mL/m²	34.2 ± 9.1	44.5 ± 12.1	<0.001ᵃ	t = −6.633
E/A ratio	0.86 ± 0.30	0.70 ± 0.31	0.002ᵃ	t = 3.386
E/e′ ratio	12.6 ± 4.0	17.4 ± 5.0	<0.001ᵃ	t = −7.190
Septal e′ velocity, cm/s	6.1 ± 1.5	5.0 ± 1.5	<0.001ᵃ	t = 4.695
Diastolic dysfunction grade, n (%)			<0.001ᶜ	χ² = 22.289
Grade I	65 (40.4%)	8 (14.5%)	—	—
Grade II	82 (50.9%)	30 (54.5%)	—	—
Grade III	14 (8.7%)	17 (30.9%)	—	—
TR velocity, m/s	2.64 ± 0.37	2.96 ± 0.48	<0.001ᵃ	t = −5.114
PASP, mmHg	36.8 ± 8.7	43.9 ± 12.4	<0.001ᵃ	t = −4.655
Elevated PASP, n (%)	33 (20.5%)	25 (45.5%)	0.001ᵇ	χ² = 13.001

In the multivariable logistic regression analysis, NYHA class III-IV was used as the dependent variable, with NYHA class I-II as the reference category. Echocardiographic parameters that demonstrated clinical relevance in the univariable analysis were entered into the multivariable model. In the analysis, E/e′ ratio, left atrial volume index (LAVI), and estimated PASP remained independently associated with greater symptom severity, whereas LVEF and LV hypertrophy were not independently associated with NYHA III-IV symptoms (Table 6). The model includes selected echocardiographic predictors. The reported odds ratios are adjusted for the other predictors included in the model; demographic characteristics and clinical comorbidities were not included as covariates.

**Table 6:** Multivariable logistic regression analysis of echocardiographic parameters associated with NYHA III–IV symptoms OR: odds ratio; CI: confidence interval; E/e′: ratio of early transmitral flow velocity to early diastolic mitral annular velocity; LAVI: left atrial volume index; PASP: pulmonary artery systolic pressure; LV: left ventricle; LVEF: left ventricular ejection fraction; mL/m²: millilitres per square metre; mmHg: millimetres of mercury; %: percentage

Echocardiographic parameter	Adjusted OR	95% CI	p-value	Test statistic
E/e′ ratio, per unit increase	1.18	1.09–1.27	<0.001ᵃ	Wald z ≈ 4.25
LAVI, per 1 mL/m² increase	1.05	1.02–1.08	0.001ᵃ	Wald z ≈ 3.35
PASP, per 1 mmHg increase	1.04	1.01–1.07	0.009ᵃ	Wald z ≈ 2.66
LV hypertrophy	1.42	0.76–2.67	0.273ᵃ	Wald z ≈ 1.09
LVEF, per 1% increase	0.96	0.92–1.00	0.061ᵃ	Wald z ≈ -1.92

## Discussion

HFpEF is a heterogeneous clinical syndrome in which significant symptoms and functional limitation may occur despite preserved LVEF. Contemporary diagnostic approaches therefore emphasize abnormalities of ventricular filling, left atrial remodeling, and pulmonary pressures rather than LVEF alone [1-3]. The present study evaluated the relationship between clinical symptom severity and echocardiographic characteristics in patients with HFpEF. The principal finding was that patients with greater symptomatic limitation, represented by NYHA functional class III-IV, demonstrated significantly greater abnormalities in E/e′ ratio, LAVI, left ventricular hypertrophy, grade of diastolic dysfunction, and estimated PASP. Furthermore, E/e′, LAVI, and PASP remained independently associated with severe symptom status on multivariable analysis. These findings demonstrate associations between greater symptom burden and echocardiographic abnormalities involving ventricular filling, left atrial remodeling, diastolic dysfunction, and pulmonary pressures. They suggest that these parameters may serve as concurrent echocardiographic correlates of symptom severity in HFpEF, although the cross-sectional design does not establish temporal or causal relationships.

The demographic characteristics of our cohort were broadly comparable with those described in Asian HFpEF populations. The mean age of the study population was 67.8 ± 10.4 years, and women constituted 53.7% of the cohort. The ASIAN-HF study, which included patients with HFpEF from multiple Asian regions, similarly demonstrated an elderly population with substantial representation of women [5]. This similarity is relevant because HFpEF is particularly prevalent among older individuals and is strongly associated with cardiometabolic risk factors. The present cohort therefore reflects the demographic characteristics commonly encountered in routine clinical practice.

A substantial burden of cardiovascular and metabolic comorbidity was observed, with hypertension being the most frequent comorbidity, followed by diabetes mellitus, obesity, chronic kidney disease, and coronary artery disease. This clustering of risk factors is characteristic of HFpEF. Hypertension contributes to increased ventricular stiffness and hypertrophy, diabetes and obesity promote metabolic and vascular dysfunction, while renal dysfunction may contribute to volume overload and impaired vascular homeostasis. Importantly, HFpEF-related symptoms are not generated exclusively by abnormalities within the left ventricle. Exercise intolerance is increasingly understood as the consequence of interacting cardiac, vascular, pulmonary, skeletal-muscle, and metabolic abnormalities [6]. Thus, the comorbidity burden in the present cohort provides an important background for interpreting the relationship between echocardiographic findings and clinical symptoms.

The most prominent association in our study was between symptom severity and E/e′ ratio. Patients with NYHA III-IV symptoms had a significantly higher mean E/e′ than those with NYHA I-II symptoms (17.4 ± 5.0 vs. 12.6 ± 4.0; p < 0.001). E/e′ also remained independently associated with severe symptoms in multivariable analysis, with an adjusted odds ratio of 1.18 for each unit increase. This finding is clinically plausible because E/e′ is an established echocardiographic marker used to estimate LV filling pressure and is incorporated into contemporary diagnostic approaches to HFpEF [2,3].

The association between elevated filling pressure and functional limitation has been demonstrated in previous studies. Holland et al. showed that exercise echocardiography can identify abnormal increases in E/e′ among patients presenting with dyspnea and preserved EF, highlighting the importance of dynamic abnormalities in LV filling pressure [7]. More recently, Obokata et al. demonstrated that low-level exercise E/e′ can provide useful information regarding pulmonary capillary wedge pressure in patients with suspected HFpEF [8]. These findings provide a physiological explanation for our observation that patients with higher resting E/e′ were more likely to have severe symptoms.

However, the relationship between E/e′ and symptoms should not be interpreted as absolute. Resting E/e′ does not capture the entire hemodynamic response to exercise, and some patients with HFpEF may have relatively modest resting abnormalities but develop substantial elevations in filling pressure during exertion [7,8]. Moreover, exercise intolerance in HFpEF is multifactorial. Impaired chronotropic response, vascular dysfunction, right ventricular abnormalities, pulmonary hypertension, obesity, anemia, and skeletal-muscle dysfunction may all contribute to symptoms [6]. Therefore, the independent association of E/e′ with NYHA class in our study suggests that elevated filling pressure is an important marker of symptomatic disease severity rather than the sole determinant of symptoms.

Left atrial remodeling was another major finding. LAVI was significantly greater among patients with NYHA III-IV symptoms than among those with NYHA I-II symptoms (44.5 ± 12.1 vs. 34.2 ± 9.1 mL/m²; p < 0.001), and LAVI remained independently associated with severe symptoms in multivariable analysis. The left atrium is chronically exposed to increased LV filling pressure in HFpEF, and sustained elevation in filling pressure produces progressive atrial enlargement and functional impairment. Consequently, LAVI may represent a marker of the chronicity of abnormal LV filling rather than a transient measure of hemodynamic status.

The clinical relevance of left atrial remodeling in HFpEF has been demonstrated previously. Donal et al. reported that the combination of increased LAVI and elevated estimated pulmonary pressure identified patients with a higher risk of death or HF hospitalization [9]. Similarly, studies of left atrial function have shown that abnormalities in atrial reservoir, conduit, and pump function are common in HFpEF and have prognostic significance [10]. Although advanced measures such as left atrial strain were not routinely available in our retrospective cohort, the significant relationship between LAVI and symptom severity supports the importance of left atrial remodeling as a marker of the clinical phenotype.

The distribution of diastolic dysfunction grades further supported the relationship between echocardiographic abnormalities and symptoms. Grade III diastolic dysfunction was substantially more frequent among patients with NYHA III-IV symptoms, whereas grade I dysfunction was more common among patients with less severe symptoms. The overall difference in diastolic dysfunction grade between the NYHA groups was statistically significant (p < 0.001). This finding is important because it demonstrates a graded relationship rather than simply an association between the presence or absence of diastolic dysfunction and symptoms.

Nevertheless, diastolic dysfunction should not be regarded as the sole mechanism underlying clinical manifestations. HFpEF is increasingly recognized as a multisystem disorder, and the severity of exercise intolerance depends on the interaction of cardiac and extracardiac abnormalities [6]. This explains why patients with apparently similar degrees of diastolic dysfunction may experience markedly different levels of functional limitation. It also emphasizes the importance of interpreting echocardiographic measurements alongside clinical characteristics and comorbidities.

PASP was another important echocardiographic correlate of symptom severity. Patients with NYHA III-IV symptoms had a higher mean PASP than those with NYHA I-II symptoms (43.9 ± 12.4 vs. 36.8 ± 8.7 mmHg; p < 0.001). PASP also remained independently associated with severe symptomatic status. These findings are physiologically consistent with the progression of HFpEF from elevated LV filling pressure to increased left atrial pressure and subsequent pulmonary venous hypertension.

Pulmonary hypertension is a common and clinically important complication of HFpEF. In a study of patients with HFpEF and pulmonary hypertension, those with pulmonary hypertension were more symptomatic and had poorer exercise capacity than patients without pulmonary vascular disease [10]. Donal et al. similarly demonstrated that the combination of enlarged LAVI and elevated estimated pulmonary pressure was associated with adverse outcomes [9]. The present findings therefore support the concept that pulmonary pressure represents an important downstream consequence of abnormal left-sided filling and may contribute directly to dyspnea and exercise limitation.

The simultaneous association of LAVI and PASP with symptom severity is particularly relevant. Chronic elevation of LV filling pressure can result in left atrial enlargement, which is followed by transmission of pressure to the pulmonary circulation. Thus, LAVI and PASP may represent different stages or consequences of the same underlying hemodynamic process. The fact that both remained independently associated with severe symptoms suggests that the clinical expression of HFpEF may be related to the cumulative effect of ventricular, atrial and pulmonary abnormalities rather than a single echocardiographic measurement.

Left ventricular hypertrophy was also more frequent among patients with severe symptoms. LV hypertrophy was present in 69.1% of patients with NYHA III-IV symptoms compared with 49.7% among those with NYHA I-II symptoms. Although this association was significant on univariate analysis, LV hypertrophy did not remain independently associated with severe symptoms in the multivariable model. This finding is clinically plausible. LV hypertrophy is an important structural substrate for HFpEF, particularly in patients with longstanding hypertension, but ventricular mass alone may not accurately reflect the current hemodynamic burden. Patients with similar degrees of LV hypertrophy may have markedly different filling pressures and pulmonary vascular responses. The stronger independent associations observed for E/e′, LAVI, and PASP suggest that these functional and downstream hemodynamic abnormalities may be more closely related to symptom severity than LV mass alone.

LVEF showed only a modest difference between the symptom groups and was not independently associated with severe symptoms. Although mean LVEF was slightly lower among patients with NYHA III-IV symptoms than among those with NYHA I-II symptoms (59.7 ± 6.1% vs. 62.0 ± 5.5%; p = 0.009), both groups had preserved systolic function. This finding reinforces one of the fundamental challenges of HFpEF: preserved LVEF does not necessarily indicate preserved cardiovascular reserve. Patients may have substantial abnormalities in diastolic function, filling pressure, and pulmonary hemodynamics despite apparently normal global systolic function [1-3].

The multivariable analysis provides an important overall interpretation of the findings. E/e′, LAVI, and PASP remained independently associated with NYHA III-IV symptoms, whereas LV hypertrophy and LVEF did not. These findings indicate that E/e′, LAVI, and PASP were associated with NYHA class III-IV symptoms after adjustment for the other echocardiographic predictors included in the model. However, because demographic characteristics and clinical comorbidities were not included as covariates, residual confounding cannot be excluded. These findings should therefore be interpreted as adjusted associations rather than evidence that these parameters independently determine symptom severity.

The high prevalence of comorbidities should also be considered when interpreting the relationship between echocardiographic parameters and symptoms. Edelmann et al. demonstrated that the contribution of comorbidities to functional impairment is particularly important in HFpEF, with obesity, coronary artery disease, renal dysfunction, atrial fibrillation, diabetes and other conditions contributing to higher NYHA class and poorer physical functioning [11]. Importantly, our study did not specifically investigate associations between individual comorbidities and echocardiographic parameters; therefore, these findings should be considered contextual rather than interpreted as direct associations demonstrated by our analysis.

The clinical importance of these observations lies in the potential utility of routinely available echocardiographic measurements. In many clinical settings, particularly resource-constrained environments, invasive hemodynamic assessment or exercise echocardiography may not be readily available. E/e′, LAVI, and PASP can generally be obtained during standard echocardiographic assessment and may provide complementary information regarding the physiological severity of HFpEF. Nevertheless, none of these parameters should be interpreted in isolation because each has recognized limitations and may be influenced by rhythm, loading conditions, age, and measurement quality [2,3,12].

Recent literature further emphasizes that exercise intolerance in HFpEF is not solely a consequence of resting diastolic dysfunction. Contemporary reviews describe a complex interaction between impaired LV relaxation, reduced systolic reserve, left and right atrial dysfunction, pulmonary vascular abnormalities, chronotropic incompetence, obesity, anemia and skeletal-muscle dysfunction [13,14]. This broader understanding is consistent with the present findings: although E/e′, LAVI and PASP were independently associated with severe symptoms, they should be viewed as markers of an important cardiovascular component of HFpEF rather than complete explanations for individual symptom burden.

Limitations of the study: Several limitations should be acknowledged. First, the retrospective cross-sectional design prevents determination of causality or temporal relationships between echocardiographic abnormalities and symptom severity. The HFA-PEFF diagnostic algorithm was not formally applied to individual patients; HFpEF classification was based on the documented clinical diagnosis and review of the available clinical records and echocardiographic findings. Second, NYHA functional class is partly subjective and may be influenced by non-cardiac factors such as obesity, pulmonary disease, anemia, and musculoskeletal limitation while resting echocardiography may not capture exercise-induced abnormalities. Third, the retrospective design relied on routinely documented clinical and echocardiographic records. Echocardiographic measurements were not independently reassessed by a second cardiologist as part of the study, so interobserver agreement could not be evaluated. The interval between clinical assessment and echocardiography varied according to the timing of patient presentation and investigation in routine clinical practice. Consequently, the clinical and echocardiographic findings may not always have represented the same point in the disease course. These factors should be considered when interpreting the observed associations between echocardiographic parameters and symptom severity. Fourth, PASP estimation depends on the quality of the tricuspid regurgitation signal and estimation of right atrial pressure. The multivariable model included selected echocardiographic predictors but did not adjust for demographic characteristics or clinical comorbidities. Consequently, residual confounding by factors that may influence both symptom severity and echocardiographic measurements cannot be excluded. The reported associations should therefore be interpreted as adjusted for the echocardiographic predictors included in the model, rather than as independent of all potential clinical confounders. Finally, resting echocardiography may underestimate abnormalities that become evident only during exercise. These limitations should be considered when generalizing the findings. Despite these limitations, the present study demonstrates a consistent relationship between clinical symptom severity and several echocardiographic markers of HFpEF severity. The strongest associations were observed for E/e′, LAVI, diastolic dysfunction grade, and PASP, while E/e′, LAVI, and PASP remained independently associated with severe symptoms after multivariable adjustment. These findings support the concept that HFpEF is not adequately characterized by preserved LVEF alone and that assessment of ventricular filling, left atrial remodeling, and pulmonary pressure provides additional information regarding clinical severity.

## Conclusions

This retrospective study demonstrates that symptom severity in patients with HFpEF is significantly associated with abnormalities in cardiac filling, left atrial remodeling, diastolic function, and pulmonary hemodynamics. Patients with more severe symptoms (NYHA class III-IV) had significantly higher E/e′ ratios, larger left atrial volume indices, greater prevalence of advanced diastolic dysfunction, and higher estimated PASPs compared with those with NYHA class I-II symptoms. Although left ventricular hypertrophy was more frequent among patients with greater symptom burden, it was not independently associated with severe symptoms after multivariable adjustment. Similarly, LVEF showed only a modest difference between symptom groups and did not independently predict symptomatic severity, reinforcing that preserved systolic function alone does not adequately characterize the clinical burden of HFpEF. On multivariable analysis, E/e′ ratio, LAVI, and PASP were associated with NYHA class III-IV symptoms after adjustment for the other echocardiographic predictors included in the model. These findings identify concurrent echocardiographic correlates of greater symptom burden but do not establish causality or independence from unmeasured clinical confounders. These findings support a multidimensional echocardiographic assessment of patients with HFpEF rather than reliance on LVEF alone. These echocardiographic parameters may provide complementary information when characterizing symptom burden in patients with HFpEF. Future prospective studies incorporating exercise echocardiography and objective measures of functional capacity may further clarify these relationships.
